# Application of Mobile Technology for Improving Expanded Program on Immunization Among Highland Minority and Stateless Populations in Northern Thailand Border

**DOI:** 10.2196/mhealth.3704

**Published:** 2015-01-14

**Authors:** Jaranit Kaewkungwal, Tawatchai Apidechkul, Kasemsak Jandee, Amnat Khamsiriwatchara, Saranath Lawpoolsri, Surasak Sawang, Aumnuyphan Sangvichean, Peerawat Wansatid, Sarinya Krongrungroj

**Affiliations:** ^1^Center of Excellence for Biomedical and Public Health Informatics (BIOPHICS)Faculty of Tropical MedicineMahidol UniversityBangkokThailand; ^2^Department of Tropical HygieneFaculty of Tropical MedicineMahidol UniversityBangkokThailand; ^3^School of Health ScienceMae Fah Luang UniversityChiang RaiThailand

**Keywords:** expanded program on immunization, EPI, hill tribes, stateless, behavioral change communication, mobile technology

## Abstract

**Background:**

Studies of undervaccinated children of minority/stateless populations have highlighted significant barriers at individual, community, and state levels. These include geography-related difficulties, poverty, and social norms/beliefs.

**Objective:**

The objective of this study was to assess project outcomes regarding immunization coverage, as well as maternal attitudes and practices toward immunization.

**Methods:**

The “StatelessVac” project was conducted in Thailand-Myanmar-Laos border areas using cell phone-based mechanisms to increase immunization coverage by incorporating phone-to-phone information sharing for both identification and prevention. With limitation of the study among vulnerable populations in low-resource settings, the pre/post assessments without comparison group were conducted. Immunization coverage was collected from routine monthly reports while behavior-change outcomes were from repeat surveys.

**Results:**

This study revealed potential benefits of the initiative for case identification; immunization coverage showed an improved trend. Prevention strategies were successfully integrated into the routine health care workflows of immunization activities at point-of-care. A behavior-change-communication package contributes significantly in raising both concern and awareness in relation to child care.

**Conclusions:**

The mobile technology has proven to be an effective mechanism in improving a children’s immunization program among these hard-to-reach populations. Part of the intervention has now been revised for use at health centers across the country.

## Introduction

### Thailand Population Statistics

The figures for minority and stateless populations, besides the population of 64 million with citizen identification in Thailand, vary between different reports, depending on the definition of the populations of interest [1]. In 2011, estimates were as high as 3.5 million non-Thai nationals living in the country [2]. Some minority groups, particularly those living in border areas, are difficult to count due to their illegal, migration, and cross-border status. It has been reported that about 30%-60% of indigenous and immigrant highlanders, so-called “hilltribes”, do not possess Thai citizenship. In a highland survey conducted in 1999, about 43% of the nearly 874,000 hilltribe population in Thailand were not recognized as Thai nationals [2,3]. In the United Nations Educational, Scientific, and Cultural Organization’s largest official household survey (Highland Peoples Survey, 2006), 37% of 63,724 hilltribe people residing in 192 border villages did not have Thai citizenship, whereas in the second survey (2010), no new estimate of unregistered hilltribes was addressed [3,4]. In Thailand (2011), there are 9761 government primary health units (PHUs), so-called Subdistrict Health Promotion Hospitals, located at each district throughout the country. Each PHU provides basic health care services and health promotion activities [5]. While the majority of stateless people face difficulties acquiring the “right to health”, including access to basic health care services [6,7], highland minority and stateless individuals along Thailand’s borders do enjoy some access to treatment and care mostly free of charge, should they wish to make the journey to the basic governmental health care facilities provided [8]. However, few nongovernmental organizations work in areas to assist these people attaining Thai citizenship to ensure their basic human rights [9].

Geographically, hilltribe settlements in Thailand are scattered over 20 western and northern border provinces of the country; however, about 90% of them reside in nine upper northern provinces. The highest proportion of the hilltribe population lived in the Chiang Rai Province, Chiang Mai Province, Mae Hong Son Province, Tak Province, and Nan Province. According to the 1995 survey, there are six major distinctive ethnic groups, each of which can be identified by distinctive costumes and languages including: Karen, Hmong, Lahu, Akha, Mein or Yao, and Lisu [10]. According to the report, there was at minimum one primary school in about half of the highland villages providing basic formal education. However, many of the older generation and new stateless migrants/cross-border hilltribe people hardly speak or understand the Thai language, but rather know a few "survival" words [10-14]. Despite most registered minority groups in Thailand having been granted Thai nationality, problems surrounding their children’s health endure, mostly due to inequalities of socioeconomic status [15]. Some stateless children are born in shelters, scattered in remote mountain regions, and never visit health facilities for treatment or care.

### The Expanded Programme on Immunisation

Thailand has adopted the Expanded Programme on Immunisation (EPI) as a national policy since 1976 [1,16,17]. Over the course of the 5 year immunization program for each child, there are 6 vaccines offered at the hospitals and PHUs across the country, including Bacillus Calmette-Guérin (BCG) vaccine for tuberculosis, HepB vaccine for hepatitis B, DTP vaccine for diphtheria-tetanus-pertussis, OPV for polio, MMR vaccine for measles-mumps-rubella, and JE vaccine for Japanese encephalitis [1]. A national survey in 2005-2006 reported that 83.3% of Thai children age 12-23 months received all 8 recommended vaccinations, while 1.3% had received none [18]. An unpublished provincial Health Office report in Thailand’s northernmost province, Chiang-Rai, showed the common health problems of highland children included pneumonia and diarrhea; incidences of measles and tetanus, and at least 3 outbreaks of diphtheria in the most remote areas, were reported in 2008-2010.

A review of the literature on the epidemiology of undervaccinated children in resource-limited settings [19-27] revealed barriers at individual, community, and state levels. These include geographic difficulties (eg, living in remote areas and too far from health care facilities), time constraints (due to parents’ work obligations/conditions during vaccination periods), the social norms and cultural structures of the community itself, and the lack of legal documents. At the global level, even though child immunization-coverage trends are positive, there remains a need to develop new mechanisms to increase immunization rates among hard-to-reach populations [28]. The use of mobile technology has been shown to improve vaccination coverage, especially among high-risk, low-income minorities [29,30]. A module integrating cell phones into routine health care systems to improve antenatal care (ANC), as well as EPI services, has already shown positive vaccination outcomes for those underserved populations in Thailand’s border areas [31].

The “StatelessVac” project was developed and implemented in Thailand-Myanmar-Laos border areas in the Chiang-Rai Province, using cell phone-based solutions with the specific purpose to provide an effective mechanism for achieving EPI coverage targets among ethnic hilltribe and stateless populations. Strategies included two system functionalities for phone-to-phone information sharing, for “identification*”* and *“*prevention*”*. “Identification” refers to valid case registration on the EPI scheme for ethnic-minority and stateless children; “prevention” is proper case management so as to receive fundamental immunizations, plus behavior-change communication measures in relation to seeking out and gaining access to health care services. The main objective of this study was to assess project outcomes regarding EPI coverage, and maternal attitudes and practices toward the immunization of their children.

## Methods

### Project Settings

**Figure 1:**
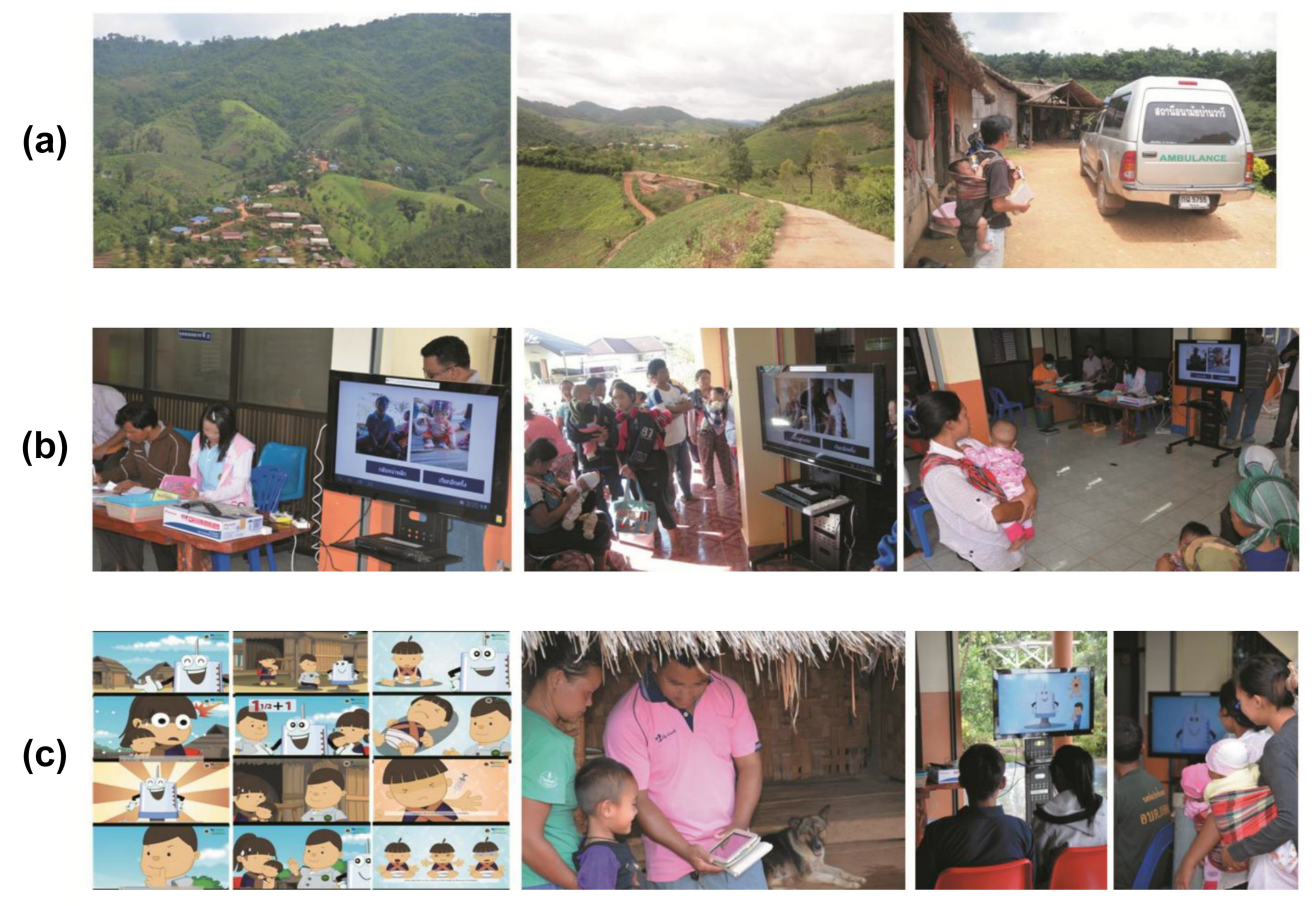
The StatelessVac project initiatives. (a) Project locations on highland; (b) picture and pronunciation for case identification; and (c) behavior change communication at health center and during routine home visit before schedule date.

The areas for project implementation consisted of all villages under the responsibility of three governmental PHUs, covering Thai citizens and both registered and unregistered highland populations. Each village was populated with a mix of 6 main highland minorities: Karen, Hmong, Mein, Akha, Lahu, and Lisu, with one immigrant minority population of Yunnan Chinese. The distances from each village to its responsible PHU ranged from 1 to 17 kilometers, with a median of 7 kilometers. Figure 1 shows examples of the different study locations.

It should be noted that in Thailand, the Ministry of Public Health has established and maintained 68 provincial hospitals, 759 district hospitals, and 28 referral (regional) hospitals, while providing 9761 primary health care services at the PHUs [5,32]. The PHUs are functioning at subdistrict level across the country. There are generally 3-5 health care staff working at each PHU, providing integrated health care that includes prevention, promotion, curative, and rehabilitation services. While health care personnel at each PHU may work with the nearby district and/or provincial hospital in terms of referral of serious cases beyond their functionalities, they mainly work with village health volunteers (VHVs). Each VHV is responsible for about 8-15 households, and home-visit activities concerning maternal and child health care is one of their monthly routine tasks. In this study, however, only 33 active VHVs, selected from 163 in the project implementation villages, were equipped with customized tools to improve immunization coverage. With the availability of the equipment to be tested, and each VHV’s workload was taken into account, other VHVs continued to work on their routine health-promoting activities with each of their households, while the 33 VHVs undertook EPI-promoting activities at their households. The other 130 VHVs were informed of these EPI activities and had no objections.

### Mobile-Technology Initiatives

In response to the call for proposal “Create Low-Cost Cell Phone-Based Solutions for Improved Uptake and Coverage of Childhood Vaccinations” by the Grand Challenges Explorations Round 7, the StatelessVac project was developed [33,34]. In order to improve vaccine delivery, particularly among underserved populations in developing countries, the challenges for which solutions were to overcome include: (1) correctly identifying an individual infant/child, and (2) connecting vaccine availability with target populations. Solutions for the challenges of infant/child identification were requested to find ways to positively record and examine the immunization history, but in many settings names and addresses are not consistently used or difficult to record even in paper-based systems, while printed identification cards can get lost or be impossible to implement. Moreover, robust biometric data for infant/child are usually difficult to validate. Another challenge was to find solutions for vaccinations that need to serve across the extended geographical area, which usually involves difficulties in travelling to receive health care services, while ensuring that the child’s family has accurate information on where and when vaccinations are to be offered. It is suggested that such an approach would consequently increase the overall vaccine coverage. The StatelessVac project was thus proposed to offer innovative approaches to address the aforementioned challenges. The challenges were also asked for "off the beaten track" and daring in premise; that was the reason for proposing to launch the initiatives among the hard to reach populations.

Mobile technology, particularly the use of smartphone systems, has already been employed as a vehicle for global health care innovation, in terms of facilitating behavior change and improving health care. The benefits of such innovations include: improved access to and quality of care, patient management, and health outcomes among underserved populations [35]. The four-pillar approach for building an effective response to statelessness, including: activities related to identification, prevention, reduction, and protection [8], can be adapted successfully by using such technology. In the StatelessVac project, two approaches were initiated: (1) case identification, and (2) disease prevention. The data/information transmission and utilization between appointed VHVs residing in each highland village and the local lowland health care center personnel responsible for area coverage was utilized. The use of tablets as part of a phone-to-phone information-sharing strategy is feasible when a telephone signal is available; other system functionalities can be performed offline. Specific information, which is shared on tablets given to the VHVs, and the standardized databases used by health care personnel at each PHU, are adapted from those recorded routine activities of original data sharing carried out on paper-based systems, when VHVs performed monthly home visits. Personal and familial information was treated confidentially within the system’s applications.

Some novel ideas for case identification include transmitting “picture” and “pronunciation” data using the phone-to-phone system from remote highland areas to lowland PHUs. Specific issues of complexity concerning the registration procedure include noncitizenship, lack of birth certificate, inconsistency of name, and unspecified residential location. Due to changes of a baby’s appearance throughout the 5 year immunization program, the picture of baby was used as a biometric data. With parental permission, pictures of each infant and its mother, taken via tablet prior to each vaccination visit, were securely transmitted to authorized health care personnel at the responsible PHU. Each hilltribe has its own dialect; most have spoken, but not written languages [10-14]. Variations in pronunciation raise even more complex issues, most hilltribe names demonstrate no connection between each speech sound, print representation, or word meaning, when listeners attempt to spell and transcribe them into the Thai language. Many newborns are given, and registered onto, the health care database with Thai names, yet many caregivers do not remember or even recognize when their infant is called out using their Thai name. This has long been a problematic public-health issue, in terms of both case registration and management, and drug and health product distribution for hilltribe communities and migrants. There are numerous and repetitive misidentified cases, with misspellings and mispronunciations of names throughout the official health care database, in both the paper- and electronic-based systems. Despite using pronunciation data for modeling of word recognition, in this project, the simple transmission of pronunciation data (baby’s name in the ethnic language spoken by its mother) was programed for use as a confirmation biometric. This was used specifically for case recognition, to correctly identify each baby due for immunization at the health care facility on the EPI schedule.

The case-identification strategy was developed and incorporated into tablet functionality. On a VHV’s tablet, after a baby was registered on the responsible PHU’s database, EPI scheduling was flagged monthly as due and/or overdue immunization(s) for each VHV’s routine home visit. Replacing monthly case management on papers between VHVs and PHU staff, the vaccination history of the infant/child was updated on each VHV’s tablet. Monthly information about EPI schedule, plus additional picture and pronunciation of each child’s name, was routinely transmitted and synchronized between VHV and responsible PHU’s tablet. On the set vaccination dates at the PHU each month, health care personnel employed pictures and pronunciation data obtained from the VHV’s tablets, by presenting the child’s picture on a television screen and calling out the child’s name in the ethnic language from the PHU’s tablet. Figure 1 shows how picture and pronunciation data were used for case identification at health care facilities on vaccination day.

Behavior change communication (BCC) and advocacy in the community is another preventive measure that was developed. Based on the United Nations Children’s Fund (UNICEF)’s guideline for communication strategy for a development program [36], the effective communication relies on the synergistic use of three strategic components including: (1) advocacy, (2) social mobilization, and (3) BCC. In the StatelessVac project, the advocacy was planned for informing and motivating leadership among health care personnel at PHUs, and VHVs to create a supportive environment to achieve a higher vaccine coverage goal using cell phone-based initiatives. Even though social mobilization was not considered as a critical part of this initiative, it was already in place under the structure of health care services of the country. The PHUs and VHVs have been actively engaging and supporting people in their responsible networking areas, performing routine home visits and promoting health care services. The BCC strategy has adopted the interpersonal approach, for example, using face-to-face communication between VHV and mother during each home visit, as well as health education presentations at the health care facility on each scheduled vaccination day. EPI-related information loaded onto VHV tablets in selectable hilltribe languages were used for recruitment of new and unregistered cases, to raise awareness and concerns about child health, as well as community advocacy about the importance of child immunization. The development of BCC content and format was based on inputs gained from EPI experts at universities and at the Ministry of Public Health, together with local experts on different hilltribe cultures. BCC scripts were translated and back-translated into multi-languages, then pretested in the villages before the final version gained approval. The BCC package currently includes 11 different animations about immunization: 10 against specific diseases, and 1 on pre and postimmunization (focus on false beliefs and culturally/locally based concepts). These animations appear in 9 languages, including 7 targeted tribal languages (Karen, Hmong, Mein, Akha, Lahu, Lisu, and Yunnan Chinese), plus 2 more languages (Thai and English) for public use. Using the case identification module, each VHV knows what vaccine(s) are due for a particular child that month; the VHV can thus show BCC animation(s) about disease-specific immunizations in the selectable ethnic language of the child’s mother. For community advocacy, BCC was presented at each PHU in random scripts and languages on vaccination day. Figure 1 shows examples of BCC activities during these home visits and at the health care facilities.

### Project Indicators and Assessment

Expected project outcomes were changes in EPI coverage, and BCC determinants over time. EPI monthly coverage data, by child age and by different type of vaccine, were collected from quarterly reports from the three PHUs to the Ministry of Public Health, submitted from January 2011 to February 2012, and from March 2012 to March 2013, before and after project implementation. BCC outcomes are based on repeat surveys from the same respondents in relation to maternal knowledge, attitudes, and practices (KAP) toward EPI vaccination. As part of the formative project evaluation, surveys were collected at months 6 and 12 after BCC was first launched. The KAP survey content was based on messages delivered via the BCC package. With due consideration of limited literacy among minorities in remote settings, the KAP questionnaire was constructed with minimum critical issues and simplicity of format. The KAP survey composes of 6 items on knowledge, 5 items on attitude, and 6 items on practice. The survey items were developed by consensus among three content experts on immunization and one on hilltribes in the study area. The survey items were piloted and revised in the nearby villages of the study areas and with different ethnics groups participating in this study. The surveys were conducted using individual interview methods by trained, designated VHVs. The VHV interviewer either spoke Thai or translated the items according to the precoded script. The analysis of the data was simply descriptive statistics.

### Ethical Considerations

Access to EPI management systems and databases is strictly controlled, and only permitted for authorized health care personnel and village health volunteers in charge of case management. Information sharing on tablets as part of the phone-to-phone mechanism maintains all crucial features of data integrity and confidentiality, mirroring the routine paper-based processes at the local health care clinics, and during routine site/home visits.

Mothers and/or children who visited the health care clinics signed no written informed consent or assent form, or when meeting with health care personnel during site/home visits; all activities were routine work performed as part of standard health care practices. Data extracted for analysis were all secondary data and summary statistics, and were not identification (ID)-linked. The authors were granted permission to use extracted data for analysis from the authorized person at the Chiang-Rai Provincial Health Office. The study protocol was reviewed and approved by the Ethics Committee, Faculty of Tropical Medicine, Mahidol University.

## Results

### Expanded Program on Immunization Coverage

**Figure 2:**
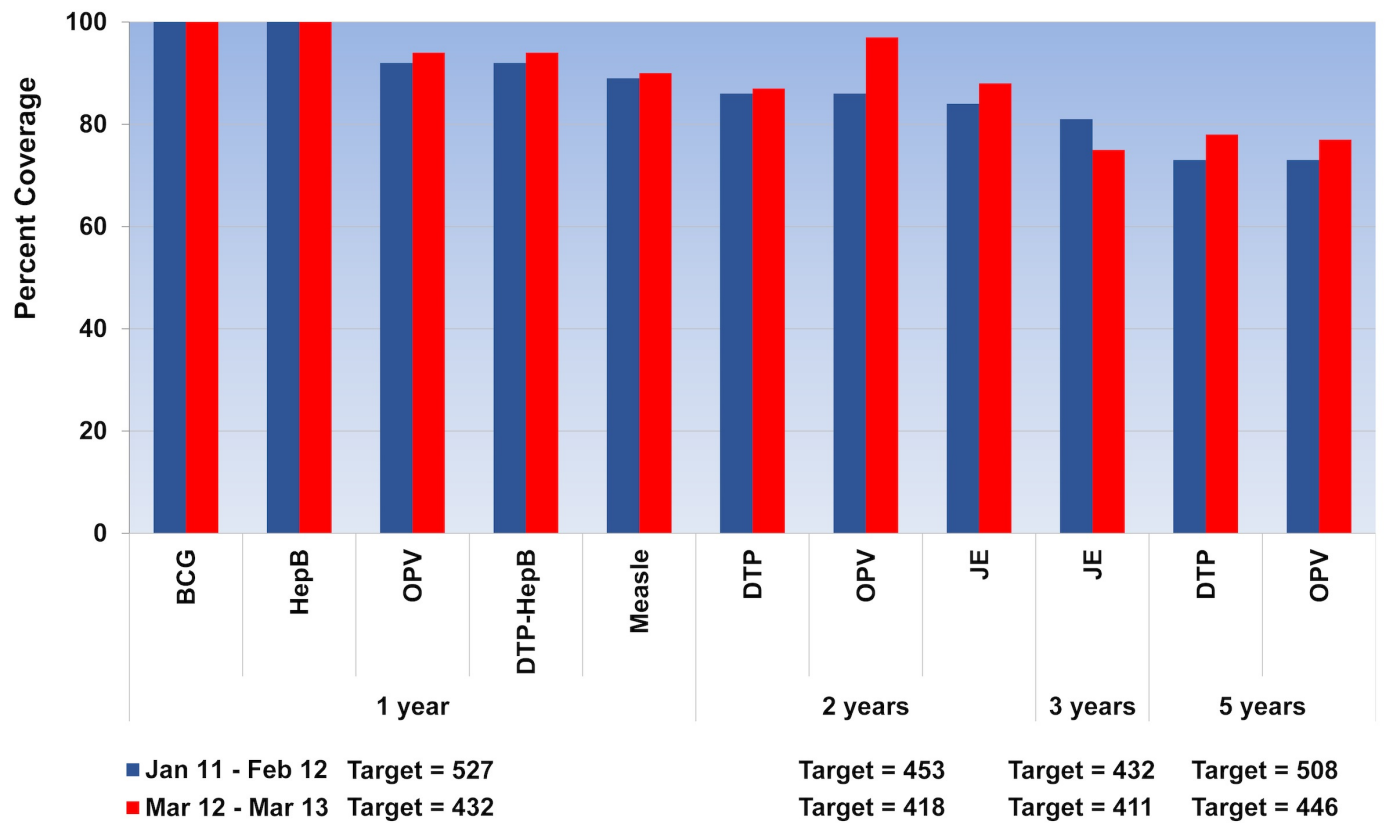
Overall immunization coverage before and after project implementation. (BCG= Bacillus Calmette-Guérin vaccine, DTP= diphtheria-tetanus-pertussis vaccine, JE= Japanese encephalitis vaccine, HepB= Hepatitis B vaccine, and OPV= oral polio vaccine).

**Figure 3:**
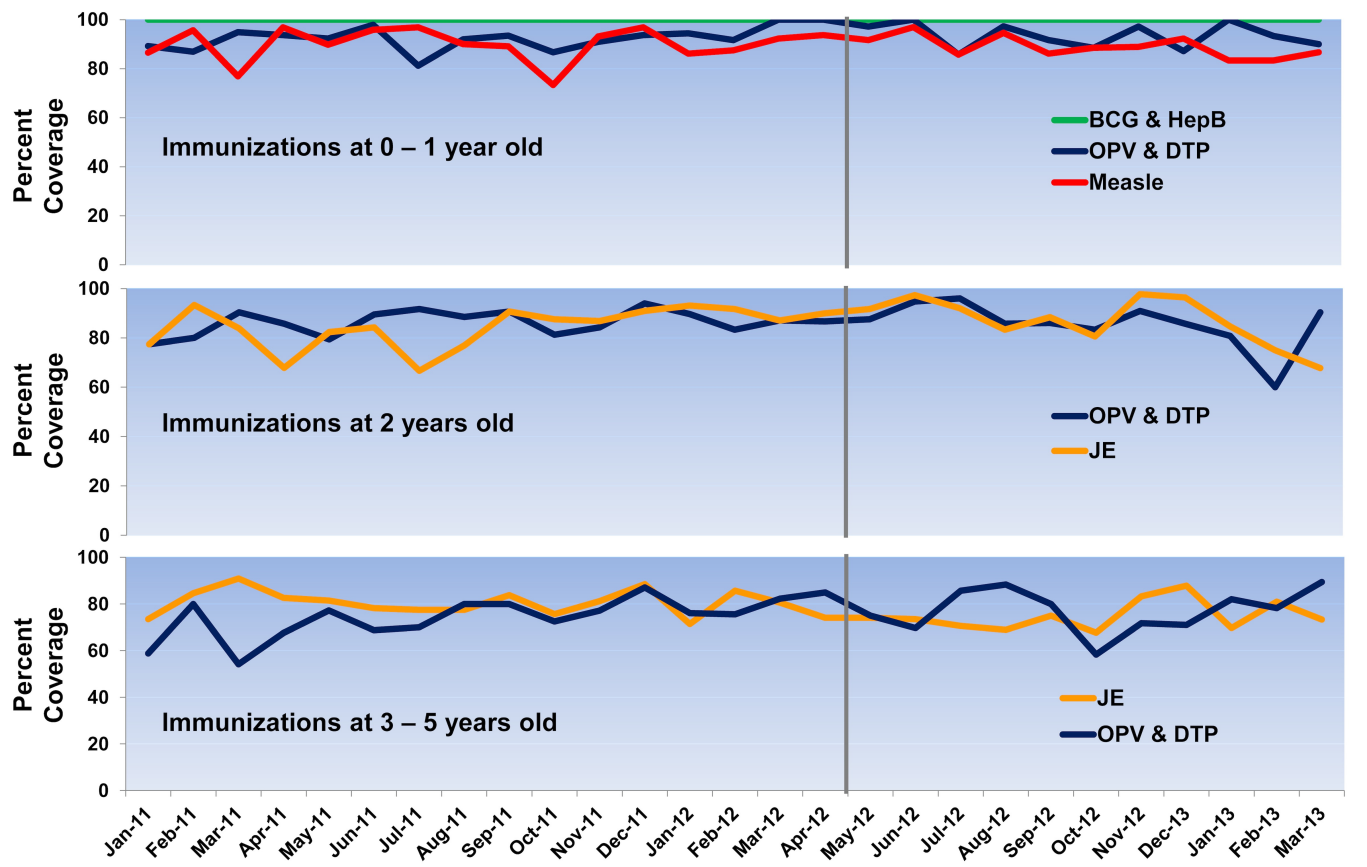
Monthly immunization rates by children ages. (BCG= Bacillus Calmette-Guérin vaccine, DTP= diphtheria-tetanus-pertussis vaccine, JE= Japanese encephalitis vaccine, HepB= Hepatitis B vaccine, and OPV= oral polio vaccine).

During the study period, a total of 3649 highland children age < 6 years were registered in the three PHUs. Immunizations for tuberculosis (BCG) and hepatitis B (HepB) have been reported at 100%, according to the records that all children were immunized at the hospital at birth. Vaccine coverage for children age 1 year showed a slight improvement after project implementation. Overall immunizations for OPV and DTP increased from 91.7% (483/527) to 94.4% (408/432), while measles increased from 89.2% (470/527) to 89.6% (387/432) (Figure 2 shows this). Fluctuations in monthly coverage suggested a slightly higher trend after tablet applications started to be used in the project areas; the minimum-maximum range for monthly immunizations of OPV-DTP changed from 81% (26/32)-98% (47/48) to 86% (30/35)-100% (36/36), and for measles vaccine 73% (33/45)-97% (31/32) to 83% (30/36)-97% (32/33) (Figure 3 shows this).

Among children age 2 years, overall coverage for OPV-DTP increased from 86.3% (391/453) to 86.6% (362/418), while JE increased from 83.9% (380/453) to 87.6% (366/418). However, the minimum-maximum range for monthly immunizations of OPV-DTP changed from 77% (24/31)-94% (31/33) to 60% (12/20)-96% (24/25), and for JE it was 67% (24/36)-93% (27/29) to 68% (21/31)-98% (43/44). Due to a change in the JE vaccine schedule for children age 3 years during the project implementation period, the overall coverage for JE decreased from 80.6% (348/432) to 75.4% (310/411), while the minimum-maximum range changed from 71% (20/28)-91% (20/22) to 68% (21/31)-88% (29/33). Among children age 4 years, the overall coverage for OPV and DTP increased from 73.2% (372/508) to 78.0% (348/446), and the minimum-maximum range for monthly immunizations changed from 54% (13/24)-87% (27/31) to 58% (21/36)-89% (17/19).

### Behavior Change Communication Determinants

Repeat KAP surveys were carried out among the 7 highland minority groups, and composed of: 32.5% (104/320) Akha, 25.3% (81/320) Lahu, 15.6% (50/320) Hmong, 11.3% (36/320) Mein, and other groups (Table 1); about 77.2% (247/320) held a Thai ID card; 12.5% (40/320) a special hilltribe status card; and 7.5% (24/320) possessed no official card at all. All respondents were mothers, with an average age of 30.3 (SD 7.9) years. Approximately 55.3% (177/320) and 40.9% (131/320) had 1-2 and 3-5 children, respectively; 58.8% (188/320) and 31.3% (100/320) had 1 and 2 children age < 6 years, respectively.

Knowledge scores, measured at months 6 and 12 after the BCC was launched, appeared to increase, but without statistical significance. The percentages of those who scored > 50% correct about a particular disease changed from 82.2% (263/320) to 88.8% (284/320) (Table 2). Regarding maternal attitudes toward immunization, about 80.9% (259/320) at month 6, and 84.4% (270/320) at month 12, displayed a highly positive attitude level (ie, a minimum 4 of 5 items ranked positively). Some with moderate attitude levels at month 6 shifted to higher positive levels. Regarding practices about preparation and having their children immunized at health facilities, most mothers changed their behaviors. At month 6, about 28.1% (90/320) of mothers reported using appropriate practices (ie, at least 5 of 6 items ranked as “always”/“never”, depending on the positive/negative items). At month 12, these practices increased significantly, to 44.1% (141/320).

**Table 1:** Characteristics of respondents in repeated KAP survey.

Characteristics		n	Percentage (N=320)
**Ethnic groups**			
	Akha	104	32.5
	Lahu	81	25.3
	Lisu	7	2.2
	Karen	1	0.3
	Hmong	50	15.6
	Mien	36	11.3
	Other	36	11.3
	Missing	5	1.5
**Age groups**			
	≤ 20 years	24	7.5
	21-30 years	148	46.3
	31-40 years	106	33.1
	≥ 41	42	13.1
**Identification holders**			
	Thai ID card	247	77.2
	Hilltribe status card	40	12.5
	Other cards	3	0.9
	Stateless status	24	7.5
	Missing	6	1.9
**Number of live children**			
	1-2	177	55.3
	3-5	131	40.9
	≥ 6	9	2.8
	Missing	3	1.0
**Number of children < 6 years**			
	1	188	58.8
	2	100	31.3
	3	11	3.4
	Missing	21	6.5

**Table 2:** KAP of mothers at months 6 and 12 after implementation of behavior change communication package.

KAP survey		Month 6 (N=320)		Month 12 (N=320)		*P* value
		n	percentage	n	percentage	
**Knowledge (6 items)**						
	≥ 80% (5-6 score)	17	5.3	12	3.8	.134
	≥ 50% (3-4 score)	246	76.9	272	85.0	
	< 50% (1-2 score)	50	15.6	30	9.3	
	None (0 score)	1	0.3	0	0.0	
	Missing	6	1.9	6	1.9	
**Positive attitude (5 items)**						
	≥ 80% (4-5 items)	259	80.9	270	84.4	.004
	≥ 50% (3 items)	40	12.6	17	5.3	
	< 50% (1-2 items)	19	5.9	24	7.5	
	Missing	2	0.6	9	2.8	
**Proper practice (6 items)**						
	≥ 80% (5-6 items)	90	28.1	141	44.1	< .001
	≥ 50% (3-4 items)	195	61.0	138	43.1	
	< 50% (1-2 items)	35	10.9	23	7.2	
	Missing	0	0.0	18	5.6	

## Discussion

### Technical Challenges Met

The technical challenge of applying information sharing between tablets of VHVs and health care personnel staff in remote areas was manageable. The telephone signals in the highland project implementation villages varied, but all data could be transmitted when a telephone signal was available, while other routine activities and data collection during each home visit were conducted offline. This proved that the innovation could be useful in hard-to-reach populations. Other research also suggests this kind of innovation can yield timely information for improving case management, delivering much higher quality, validity, and reliability [35,37]. In a similar study using smartphones to promote maternal and child health, mobile technologies were reported as an effective platform via provision of 6 core functionalities: (1) informing stakeholders, (2) training health providers, (3) enabling more accurate and precise self-monitoring, (4) delivering prompt reminders/supportive messages, (5) supporting effective service delivery over time, and (6) alerting care providers to specific cases [38]. In terms of the monitoring assessment carried out during the course of the project, the tablet application appears to have been well accepted by health care providers (ie, PHU personnel and VHVs) and by highland people in the villages. However, it should be noted that there was no formal assessment on acceptance of the technology conducted in this study; the acceptance was simply based on unstructured interviews with all health care providers in the study areas and with some random villagers during project monitoring of VHVs’ home visit activities. At the start of project implementation, however, there were minor changes in the information sharing process, and customization of application displays corresponding with the needs of VHVs and mothers.

In 2011, Thai nationwide statistics indicated that vaccine coverage rates were > 90%: 99% for BCG, DTP, and OPV; and 98% for measles and HepB [39]. But as found in this study, the rates were slightly lower for highland minority and stateless populations. In this study, slight improvement on vaccine coverage rates was shown; this could be partially explained by the study having intensified awareness in the community area. Such improvement was also observed during the first initiative to use cell phones for boosting ANC/EPI activities among migrants on the western border of Thailand [31]. In the report on updates of evidence-based strategies for children’s health and well-being [40] regarding the effectiveness of both patient-oriented intervention (including client reminder/recall system and education) and system-oriented intervention (including home visiting and case management), the findings of several studies over the decade revealed that client reminder/recall interventions could increase immunization rates by 5% to 20%, and clients who received home visits had significantly higher completed immunization rates. Overall coverage of different vaccines in this study showed increment in the range of 1% to 5%. The increasing rates appear to confirm the notation on stability in coverage once vaccination rates increase above a certain threshold, such that the increase in either developed or developing countries tends to taper off after 85% [41,42]. It was suggested, however, that good performance in immunization strategies for health care practices should be measured as achieving and maintaining coverage rates necessary to assure herd immunity of which the lower bounds was between 75% to 90% for most vaccine preventable diseases [42]. As shown in this study, the observed immunization rates in the study populations are above the lower-bounds and moving toward higher coverage rates.

As suggested in the literature, nonimmunization or lower immunization rates could be partly attributable to the complexity of individual, community, and legal factors involved. The most common explanations about this outcome concern competing priorities in child immunization, working longer hours due to poverty, and being socially and legally alienated [20,23,43]. Studies of trends in disparities in complete immunization indicate other critical factors, including low parental education levels, availability and ease of access to health care facilities, and commuting difficulties for rural and remote populations [43-45]. A main challenge of this project was the distance from the highland villages to the responsible PHUs. PHU personnel and VHVs reported concerns about distance, and also about the use of bad roads and paths, especially during the rainy season. Interestingly, documents from the grey literature for the World Health Organization (WHO) have suggested demographic or sociological characteristics are underlying or secondary determinants; that a primary factor is lack of communication, and efforts to distribute information promoting immunization [43]. It has been suggested that vaccine-related communications and parent-provider interactions are critical, such that effective interaction would address concerns and motivate parents toward completing immunization schedules. Poor communication, in contrast, introduces feelings of rejection and dissatisfaction [46]. In this project, trained VHVs are considered key players, since they are the ones who actually work in the villages, encouraging those already registered, as well as newcomers from remote areas, to engage with EPI efforts. Despite VHVs being members of the 7 tribes, not even they can speak all languages in their areas of responsibility. They need to be able to maintain good relationships, and be able to open clear communication lines between the PHUs and villagers. They also need to understand basic concepts about vaccination processes, as well as national and local EPI strategies and management. Training in new ways to conduct their duties in the villages, as well as using new technologies, is important for the success of this initiative. Refresher courses on updated versions of the developed applications are also important, not only for VHVs, but PHU staff as well.

Differences in culture and beliefs in relation to the health care-seeking behaviors of hilltribe peoples represent an ongoing challenge. The BCC animations with selectable languages have been implemented and accepted by all stakeholders. As part of the development of a selectable language BCC, the script translation into 7 ethnic languages was quite a challenge. While many common and technical words are equivalent to Thai or English, sometimes no words had precisely the same meaning. Moreover, some words have different spellings and pronunciations, even within the same tribe. However, posttesting translations and back-translations and piloting their use among several tribal groups achieved a solution.

In analyzing the postlaunch success of BCC in the community, the KAP survey revealed that the mothers had good knowledge, a positive attitude, and employed proper practices at completion of the 6 month survey, and demonstrated even higher levels at the 12 month follow-up. In this study, even though knowledge of diseases under the EPI scheme measured at 2 time points were not statistically significant; the overall percentage of correct answers was higher at 12 months. As shown by studies of factors associated with completing immunization in vulnerable populations, the primary goal of EPI activities and interventions should be strengthening communication and raising awareness in the community; inadequate knowledge regarding the objectives and importance of immunization demonstrably leads to low vaccine coverage [47,48]. The BCC in different languages appears successful in increasing positive attitudes toward immunization, and also in encouraging a shift toward proper practices in terms of prompting and preparing for EPI activities. Such changes can be said to have contributed to the higher percentages of vaccine coverage seen in the project areas. As evidenced in other studies, a positive attitude and proper practices in relation to applying knowledge are significant predictors of improved maternal health-seeking behaviors toward child health [49-51]. However, it is well recognized that one of the weaknesses of the KAP study is that it is difficult to draw out a policy implication from study outcomes. The challenge of KAP outcomes after implementation of BCC regarding EPI in this study is particularly about its potential impact on promoting the awareness of the importance of the EPI program among illegal or underserved populations. The health care providers at remote areas in this study have been informed with the study results and continued using the BCC in their responsible communities. The BCC and the study results were also presented to authorities of the Department of Disease Control at the Thai Ministry of Public Health, and the BCC packages developed in this study were consequently considered and used as part of the department EPI promotion program.

### Conclusions

To meet the challenge of attaining their maternal and child health Millennium Development Goals, the WHO and UNICEF have recommended countries to implement a central strategy of immunizing hard-to-reach infants and other age groups by focusing more on work carried out at district level [52]. This study reveals potential benefits of the initiative; mobile-technology applications for case ID and for behavior-change communication strategies, integrated into the routine EPI work at point-of-care through subdistrict health care systems. Immunization coverage has shown an improved trend among vulnerable populations who remain underserved and who often receive limited recognition. The BCC package appears beneficial and contributes to the overall success of the project in raising child-care awareness and concern. The BCC content used in this project is currently being revised, due to changes in the immunization schedule. The package is also expanding to cover another language, Burmese, which over a million migrants working in urban-rural settings and border areas of the country use to communicate. The BCC version for highland populations was disseminated to border area health centers in northern Thailand; the revised version for Thai and Myanmar migrants will be used all across the country at health centers under the Thai Ministry of Public Health. The BCC packages are now available as Google applications, which can be downloaded from the attachment of this manuscript and/or Google Play Store on android system (application title, Vaccine EPI).

## Multimedia Appendix 1

Example of edutainment animation on expanded programme on immunization (EPI) in each dialect languages.

## Multimedia Appendix 2

Google application of the BCC package in Thai version.

## Multimedia Appendix 3

Google application of the BCC package in English version.

## Multimedia Appendix 4

Google application of the BCC package in Myanmar version.

## Multimedia Appendix 5

Google application of the BCC package in Akha version.

## Multimedia Appendix 6

Google application of the BCC package in Yunnan Chinese version.

## Multimedia Appendix 7

Google application of the BCC package in Hmong version.

## Multimedia Appendix 8

Google application of the BCC package in Karen version.

## Multimedia Appendix 9

Google application of the BCC package in Lahu version.

## Multimedia Appendix 10

Google application of the BCC package in Lisu version.

## Multimedia Appendix 11

Google application of the BCC package in Yao version.

## Abbreviations

ANC: antenatal care
BCC: behavior change communication
BCG: Bacillus Calmette-Guérin vaccine
DTP: diphtheria-tetanus-pertussis vaccine
EPI: Expanded Programme on Immunisation
HepB: Hepatitis B vaccine
ID: identification
JE: Japanese encephalitis vaccine
KAP: knowledge, attitudes, and practices
OPV: oral polio vaccine
PHU: primary health unit
UNICEF: United Nations Children’s Fund
VHVs: village health volunteers
WHO: World Health Organization
